# Adipokinetic Hormones and Their Receptor Regulate the Locomotor Behavior in *Tribolium castaneum*

**DOI:** 10.3390/insects16040407

**Published:** 2025-04-12

**Authors:** Rui-Han Lu, Xu-Dong Pang, Shuang-Qin Wen, Guy Smagghe, Tong-Xian Liu, Shun-Hua Gui

**Affiliations:** 1 Guizhou Provincial Key Laboratory for Agricultural Pest Management of the Mountainous Region, Institute of Entomology, College of Agriculture, Guizhou University, Guiyang 550025, China; lrh8032025@163.com (R.-H.L.); jsgz412@163.com (X.-D.P.); 18885644922@163.com (S.-Q.W.); guysma9@gmail.com (G.S.); tx.liu@gzu.edu.cn (T.-X.L.); 2 Institute of Plant Health and Medicine, Guizhou University, Guiyang 550025, China

**Keywords:** neuropeptide, RNAi, locomotor behavior, red flour beetle

## Abstract

The regulation of locomotor behavior is essential for insects to perform their life activities. The central nervous system plays a pivotal role in modulating physiological behaviors, particularly movement, with neuropeptides serving as key modulators of these processes. Among these, adipokinetic hormone (AKH) was originally identified in insects as a neurohormone involved in lipid mobilization. This study investigates the functional role of AKHs (AKH1 and AKH2) and their receptor (AKHR) in regulating locomotion in the red flour beetle, *Tribolium castaneum*. Using functional calcium reporter assays, we demonstrated that AKHR is activated by two mature AKH peptides from *T. castaneum*, with half-maximal effective concentrations (EC_50_) falling within the nanomolar range. Gene expression analysis confirmed the presence of *AKH1* and *AKH2* transcripts in the brain, while *AKHR* expression was localized to the fat body and carcass. The silencing of *AKHs* or *AKHR* through RNA interference resulted in a significant reduction in both movement distance and duration. Collectively, these findings highlight the regulatory influence of AKH/AKHR signaling in locomotor activity in *T. castaneum*, thereby advancing our understanding of the molecular mechanisms underlying locomotor control in this economically important insect species.

## Simple Summary

The insect nervous system regulates locomotor behavior through the use of specialized signaling molecules known as neuropeptides. One such neuropeptide, adipokinetic hormone (AKH), is known to help insects use energy from fats. In this study, we explored how AKH and its receptor (AKHR) affect locomotion in the red flour beetle, Tribolium castaneum, a pest that damages stored grains. We found that AKH strongly activates its receptor, triggering responses at very low concentrations. The genes for AKH were active in the brain, while the receptor was found in the fat-storing tissue. When we reduced AKH or AKHR levels using RNA interference techniques, the beetles moved much less, showing that this signaling pathway is crucial for locomotion. These findings help us understand how insects control their activity, which could lead to better ways to manage pest insects in agriculture and food storage.

## 1. Introduction

The locomotor behavior of insects is fundamental to their survival, impacting essential behaviors such as searching for food, finding mates, escaping predators, and adapting to changing environments. Coordinated movement requires the seamless integration of internal physiological states and external sensory inputs in insects [1,2]. Insect neuropeptides, as small intercellular signaling molecules primarily secreted by the central nervous system, play an integral role in regulating various physiological functions, including locomotion, by interacting with their specific receptors [3,4]. Previous studies have identified neuropeptide–receptor systems being involved in controlling the locomotor behavior in insects. For instance, neuropeptide F had suppressive effects on phase-related locomotor activity in *Locusta migratoria* [5]. Silencing the diuretic hormone receptor resulted in reduced mean velocity in *Drosophila melanogaster* [6]. Moreover, the tachykinin and short neuropeptide F pathways in the central complex have specific roles in the fine-tuning of locomotor activity in adult *D. melanogaster* [7]. Despite these findings, limited attention has been given to the regulatory impact of neuropeptide–receptor systems on locomotor behavior in only a small number of insect species.

Adipokinetic hormone (AKH), a neuropeptide initially isolated from locusts as a neurohormone that facilitates lipid mobilization during flight, is now known to be widespread across insect species [8]. It is documented that AKH plays a vital role in regulating energy mobilization, impacting various physiological processes such as starvation responses, stress adaptation, development, and reproductive capacity [9,10,11,12,13]. These functions are mediated through adipokinetic hormone receptors (AKHRs) that share similarities with vertebrate gonadotropin-releasing hormone receptors [14].

*Tribolium castaneum*, commonly known as the red flour beetle, is a widespread pest of stored grains, flour, cereals, and other dried food products, causing significant economic damage through contamination. Both the larvae and adults feed on these materials, thriving in warm, dry environments typical of storage facilities. Beyond its role as a pest, *T. castaneum* is a valuable model organism in scientific research. Its short life cycle, ease of lab rearing, and fully sequenced genome make it ideal for studying development, genetics, evolution, and pest control strategies. Notably, it is also used to explore questions in insect biology that complement research on other insects like *D. melanogaster* [15].

In this study, we researched the regulation of the locomotor behavior in *T. castaneum* by AKHs and their receptor. Using molecular profiling and calcium mobilization assays, we comprehensively characterized the *T. castaneum* AKHs (Tc-AKHs) and their receptor (Tc-AKHR), thereby confirming the receptor functional activity of the receptor. Through RNA interference (RNAi), we further demonstrated that silencing the expression of *Tc-AKHs* or their receptor significantly altered locomotor patterns in adult beetles. Collectively, these findings provide valuable insights into the neuromodulatory mechanism underlying insect locomotion.

## 2. Materials and Methods

### 2.1. Insect Rearing and Cell Line Maintenance

The *T. castaneum* colony was reared in a controlled growth chamber under specific environmental conditions of 30 ± 1 °C temperature, 70 ± 5% relative humidity, and continuous darkness [16,17]. The insects were provided with a diet consisting of whole wheat flour and brewer’s yeast in a 19:1 ratio. Concurrently, the CHO-WTA11 cell line (generously provided by Professor Jiang Hongbo from Southwest University, Chongqing, China) was cultured in a high-glucose Dulbecco’s Modified Eagle Medium (DMEM; Sigma, Taufkirchen, Germany) supplemented with 10% fetal bovine serum (FBS; Gibco, Jenks, OK, USA). The cells were maintained in a 5% CO_2_ incubator to ensure optimal growth conditions.

### 2.2. Sequence Analysis of Tc-AKHR and Its Ligands in T. castaneum

Previous studies enabled the identification of the pre-mRNA sequences of *Tc-AKH1* (NP_001107797.1) and *Tc-AKH2* (NP_001107818.1), as well as the *Tc-AKHR* sequence (NM_001083340.1), through the NCBI database. The transmembrane domains of *Tc-AKHR* were predicted using the TMHMM version 2.0. The corresponding amino acid sequences were derived via ExPASy and subsequently aligned with homologous sequences using NCBI’s blastp tool. Phylogenetic analysis was performed using MEGA 5.1, while sequence alignment was conducted with Jalview 2.11. Signal peptides within the *Tc-AKH* precursor were predicted using the SignalP server, and C-terminal motifs were visualized using WebLogo.

### 2.3. Structural Modeling and Molecular Docking

The amino acid structures of Tc-AKHs were drawn using ChenDraw (v21.0), while homology modeling of Tc-AHKR was conducted via the Alphafold3 tool to generate a three-dimensional structural model. Molecular docking simulations were performed using the CDDOCKER module in Discovery Studio 21.0. The interaction patterns between Tc-AKHs and Tc-AKHR were visualized and analyzed using PyMOL v.3.11.

### 2.4. Functional Calcium Reporter Assays

The open reading frames (ORFs) of *Tc-AKHR* were cloned into the pcDNA 3.1 (+) expression vector (Tsingke, Beijing, China) for heterologous expression in CHO-WTA11 cells, which served as the reporter system for *Tc-AKH*-induced calcium mobilization. *Tc-AKH* peptides were chemically synthesized based on high-performance liquid chromatography (HPLC) and mass spectrometry (MS) analyses (Zoonbio, Nanjing, China). For GPCR assays, a mixture of expression vectors, containing *Tc-AKHR* (2.5 μg) and apoaequorin in a 1:1 ratio, was transfected into CHO cells using TransIT^®^-LT1 (Mirus, Madison, WI, USA). The transfected cells were harvested, resuspended in a serum-free high-glucose medium, and incubated with coelenterazine h (Thermo Fisher Scientific, Waltham, MA, USA) under dark conditions. Synthetic *Tc-AKH* peptides, prepared in 10-fold serial dilutions ranging from 2 pM to 20 μM, were added to an opaque 96-well plate. Luminescence was quantified using a SpectraMax L chemiluminescence microplate reader (Molecular Devices, San Jose, CA, USA) [18]. All experiments were conducted in three biological replicates.

### 2.5. Gene Expression in Different Tissues

Tissue-specific gene expression profiles of *Tc-AKHR* and *Tc-AKHs* were obtained from the Beetle Atlas database (https://motif.mvls.gla.ac.uk/BeetleAtlas/). Origin 2024 was employed to analyze and visualize the differential expression patterns across various tissues.

### 2.6. Total RNA Isolation and cDNA Synthesis

For total RNA isolation, three adult beetles were placed in RNase-free centrifuge tubes, flash-frozen in liquid nitrogen, and homogenized using a disposable tissue grinding rod under continuous liquid nitrogen cooling. Total RNA was extracted from whole-body samples using the Eastep^®^ Super Total RNA Extraction Kit (Promega, Madison, WI, USA). RNA integrity was assessed on 1% agarose gels, and purity was determined using a Nanodrop 2000 spectrophotometer (Thermo Fisher Scientific, USA). Subsequently, 1 µg of total RNA was reverse-transcribed into cDNA using the StarScript Pro All-in-one RT Mix with gDNA Remover (GenStar, Beijing, China), following the manufacturer’s protocol.

### 2.7. Synthesis of Double-Stranded RNA

The synthesis of double-stranded RNA (dsRNA) targeting specific genes (dsEGFP, dsAKHR, dsAKH1, and dsAKH2) was initiated by generating templates through PCR amplification. Gene-specific primers, incorporating the T7 polymerase promoter sequence at their 5′ ends (Table S1), were used to amplify the target gene fragments. The resulting PCR products were purified and subsequently employed as templates for dsRNA synthesis using the TranscriptAid™ T7 High Yield Transcription Kit (Thermo Fisher Scientific, USA), following the manufacturer’s instructions. Post-synthesis, the dsRNA was purified via phenol/chloroform extraction and ethanol precipitation and then resuspended in DEPC-treated water. The concentration of the synthesized dsRNA was quantified using a Nanodrop 2000 spectrophotometer (Thermo Fisher Scientific, USA) at 260 nm, and its integrity was verified via electrophoresis on 1% agarose gels.

### 2.8. Quantitative Reverse Transcription PCR

Quantitative real-time PCR (qRT-PCR) was conducted using the CFX Opus Real-Time PCR System (Bio-Rad, Hercules, CA, USA). Each reaction mixture consisted of 2 µL of cDNA (200 ng/µL), 1 µL each of forward and reverse gene-specific primers (10 µM), 6 µL of DEPC-treated water, and 10 µL of 2×RealStar Fast SYBR qPCR Mix (GenStar, China). The thermal cycling protocol included an initial denaturation step at 95 °C for 2 min, followed by 39 cycles of 95 °C for 15 s and 60 °C for 30 s. Gene expression levels were quantified in duplicate and normalized to the internal control gene, *T. castaneum* ribosomal protein S3 (*Tcrp3*) [19]. Primer sequences for the target genes are provided in Table S1. Relative gene expression was calculated using the 2^−∆∆CT^ method [20].

### 2.9. RNA Interference and Movement Analysis

Three-day-old adult *T. castaneum* beetles were anesthetized with CO_2_ and immobilized on double-sided tape for microinjection. Approximately 300 ng of dsRNA was injected into the ventral side of the first abdominal segment using a microinjection system (WPI-PV830, World Precision Instruments, Sarasota, FL, USA) equipped with a glass capillary needle [21]. Beetles injected with dsEGFP served as the control group. Following injection, all adults were maintained under identical conditions for 48 h prior to analysis.

For movement assessments, all tests were conducted at room temperature. Groups consisting of three adults each from the treatment (dsAKHR, dsAKH1, and dsAKH2) and control (dsEGFP) groups were transferred to Petri dishes with a diameter of 125 mm. Their motion was recorded for 1 min using a camera (Sony FDR-AX60, Tokyo, Japan) under controlled conditions. The recorded videos were analyzed using the EthoVision XT 17.5 (Noldus, Wageningen, The Netherlands) to quantify movement parameters and generate trajectories. Subject detection was achieved with gray scaling. Thresholds for “moving” and “resting” states were defined as 5 mm/s and 1 mm/s, respectively, based on slight modifications of previously established criteria [17]. For the determination of the active time, the tracking target was divided into a certain number of pixels according to the automatic setting of the software. When 5–60% of the pixels changed, it was considered to be in an active state.

### 2.10. Statistical Analysis

Statistical analysis began with the Shapiro–Wilk test to evaluate the normality of the datasets. For data conforming to a normal distribution (Shapiro–Wilk test: *p* > 0.05), an independent Student’s *t*-test was used to compare mean values of gene expression levels and other parameters between the RNAi-treated and control groups. In cases where data deviated from normality (Shapiro–Wilk test: *p* ≤ 0.05), the nonparametric Mann–Whitney U test was applied. All results are presented as the mean ± SEM from five or more independent experiments. A *p*-value of <0.05 was considered statistically significant.

## 3. Results

### 3.1. Sequence Analysis and Molecular Characterization of AKHs and AKHR in T. castaneum

**Figure 1:**
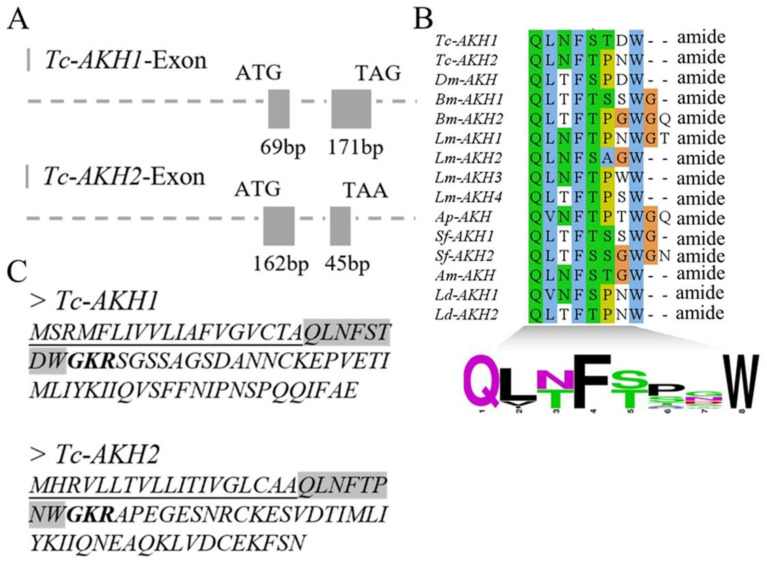
Gene structures and deduced amino acid sequences of *AKHs* in *T. castaneum*. (**A**) Gene structures of *Tc-AKH1* and *Tc-AKH2*. Exons are represented by boxes and introns by lines. (**B**) Sequence alignment of mature *AKH* peptides from *T. castaneum*, *D. melanogaster*, *B. mori*, *L. migratoria*, *A. pisum*, *S. frugiperda*, *A. mellifera* and *L. decemlineata*. The calculated sequence logo is shown at the bottom. (**C**) Deduced amino acid sequences of *Tc-AKHs*. Putative signal peptides are underlined; mature peptides are shaded in gray, and predicted amidation signals with dibasic cleavage sites (KR) are shown in bold.

The full-length pre-mRNA sequences of *Tc-AKH1* and *Tc-AKH2* each consist of two exons. The exons in *Tc-AKH1* are 69 bp and 171 bp in length and encode a protein of 73 amino acids, while those in *Tc-AKH2* are 162 bp and 45 bp in length and encode a 68-amino acid protein, respectively (Figure 1A,C). Notably, the first 19 amino acid residues in both *Tc-AKH1* and *Tc-AKH2* are predicted to function as secretory signal peptides, followed by a conserved mature peptide that ends with a predicted amidation site and a dibasic cleavage signal (GKR) (Figure 1C). When compared to the mature AKH peptide sequences of *T. castaneum*, *D. melanogaster*, *Bombyx mori*, *L. migratoria*, *Acyrthosiphon pisum*, *Spodoptera frugiperda*, *Apis mellifera*, and *Leptinotarsa decemlineata*, it was observed that the AKH peptides are highly conserved and displayed the following characteristic motif: QxxxxxxWamide (Figure 1B).

**Figure 2:**
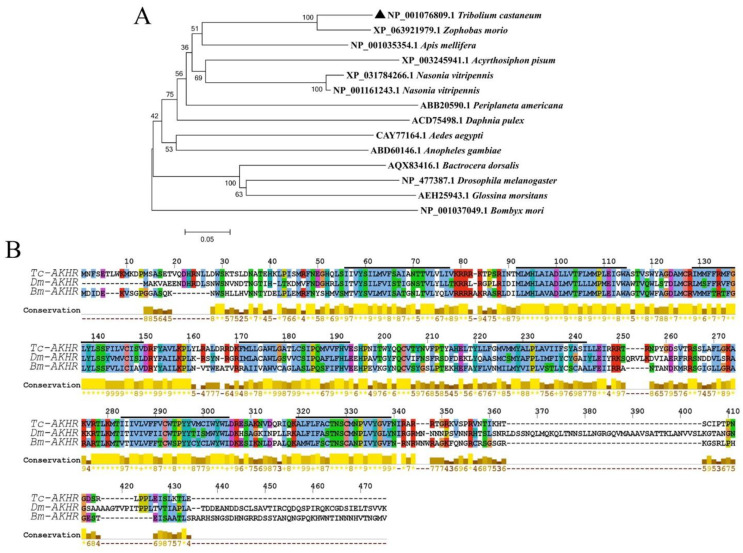
Amino acid sequence alignment and cluster analysis of *AKHR* in *T. castaneum*. (**A**) Evolutionary tree analysis of *Tc-AKHR* (neighbor-joining). The insect *AKHR* protein sequences of *T. castaneum*, *Z. morio*, *A. mellifera*, *A. pisum*, *N. vitripennis*, *P. americana*, *D. pulex*, *A. aegypti*, *A. gambiae*, *B. dorsalis*, *D. melanogaster*, *G. morsitans*, and *B. mori* were selected to construct the evolutionary tree. *Tc-AKHR* was assigned with “▲”. (**B**) Comparative amino acid sequence alignment of *T. castaneum*, *D. melanogaster*, and *B. mori AKHR* sequences.

The *Tc-AKHR* mRNA features a putative ORF of 1137 nucleotides that encodes a protein consisting of 378 amino acids. *Tc-AKHR* is a membrane-bound protein with seven transmembrane domains, consistent with its classification as a G protein-coupled receptor (GPCR). Comparative analysis with AKHR sequences from *D. melanogaster* and *B. mori* revealed a high degree of conservation. The *Tc-AKHR* sequence shares 52.77% similarity with *D. melanogaster AKHR* and 59.73% similarity with *B. mori AKHR*, particularly in the amino acid sequence of the transmembrane domains (Figure 2B).

Phylogenetic analysis was further conducted using MEGA5.1 to clarify the evolutionary relationship of *Tc-AKHR* within the arthropod phylum. Using the neighbor-joining method, a gene cluster analysis was performed, which showed that the *Tc-AKHR* from *T. castaneum* (order: Coleoptera) is most closely related to the AKHR from *Zophobas morio* (order: Coleoptera), followed by that from *A. mellifera* (order: Hymenoptera) (Figure 2A).

### 3.2. Activity Determination of Tc-AKHs on Tc-AKHR

**Figure 3:**
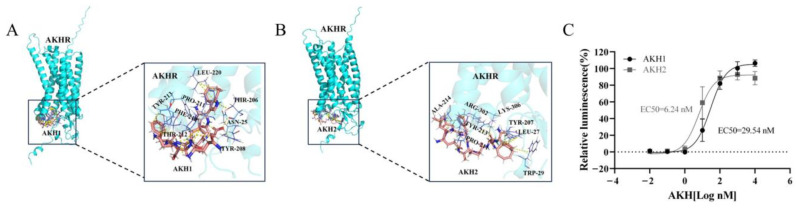
(**A**,**B**) *Tc-AKH1* and *Tc-AKH2* docked to *Tc-AKHR*. The hydrogen bond is represented by a yellow dotted line; the three letters are the abbreviation of the amino acid. and the number is the amino acid number of the receptor. (**C**) Dose–response curves and EC_50_ values of *Tc-AKH1* and *Tc-AKH2*, tested on *Tc-AKHR* expressed in CHO-WTA11 cells.

Docking simulations revealed that both Tc-AKH1 and Tc-AKH2 bind to specific docking sites on Tc-AKHR, with each ligand interacting with a distinct set of eight amino acids on the receptor (Figure 3A,B). These sites are crucial for the binding of Tc-AKHs to Tc-AKHR.

To evaluate the interaction between *Tc-AKHs* and *Tc-AKHR*, heterologous expression of *Tc-AKHR* was carried out in CHO-WTA11 cells, followed by calcium reporter assays. The results indicated that the CHO cells expressing *Tc-AKHR* were successfully activated by chemically synthesized *Tc-AKHs*. Among the two ligands, *Tc-AKH1* demonstrated a higher sensitivity, with an effective concentration (EC_50_) of 6.24 nM, compared to *Tc-AKH2*, which exhibited an EC_50_ of 29.54 nM (Figure 3C).

### 3.3. Expression of Tc-AKHs and Tc-AKHR in Different Tissues of T. castaneum

**Figure 4:**
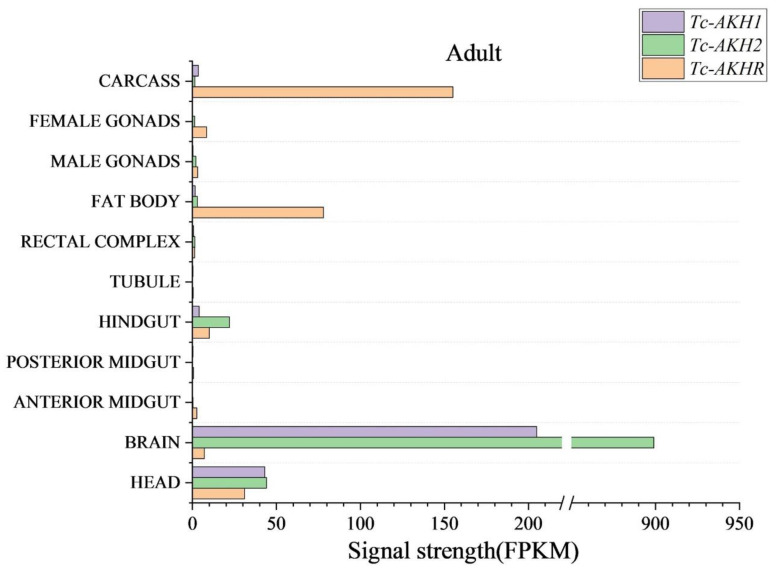
The relative expression of *Tc-AKHR*, *Tc-AKH1*, and *Tc-AKH2* in different adult tissues of *T. castaneum*. Data were obtained from the Beetle Atlas database.

To investigate the tissue-specific expression patterns of *Tc-AKHR* and *Tc-AKHs*, transcript levels were assessed across various tissues in adult *T. castaneum* according to data from the Beetle Atlas database. The expression of *Tc-AKHR* was found to be higher in the fat body and carcass. The two *Tc-AKHs* exhibited a very high expression in the brain (Figure 4).

### 3.4. Effects of Tc-AKH and Tc-AKHR Knockdown on Locomotion

**Figure 5:**
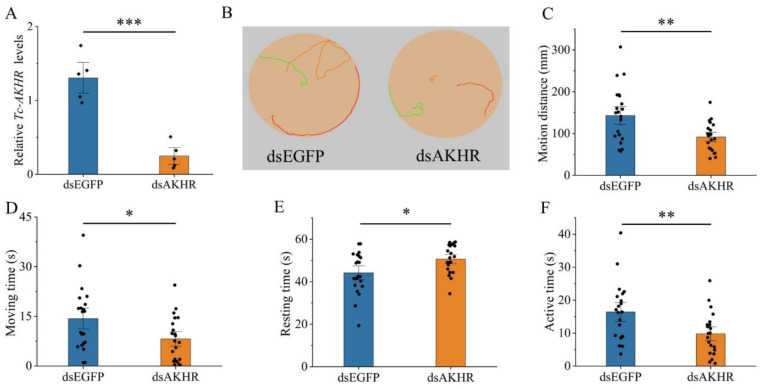
Effect of dsAKHR injected into three-day-old adults on the gene transcript levels and motion situation of *Tc-AKHR*. The data were analyzed using Student’s *t*-test or the Mann–Whitney U test. Date are means ± SE. * *p* < 0.05, ** *p* < 0.01, and *** *p* < 0.001. (**A**) Relative expression levels of *Tc-AKHR* at 48 h after injection with dsAKHR; dsEGFP was used as a control. (**B**) Movement trajectory after injection with dsAKHR. (**C**) Changes in motion distance. (**D**) Changes in moving time. (**E**) Changes in resting time. (**F**) Changes in active time.

**Figure 6:**
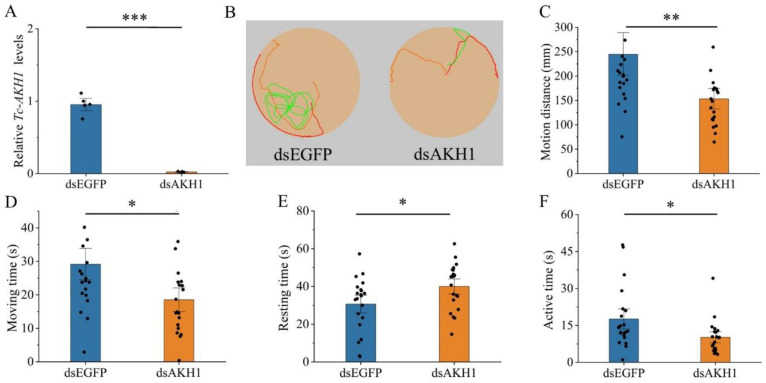
Effect of dsAKH1 injected into three-day-old adults on the gene transcript levels and motion situation of Tc-AKH1. Date are means ± SE. * *p* < 0.05, ** *p* < 0.01, and *** *p* < 0.001. (**A**) Relative expression levels of Tc-AKH1 at 48 h after injection with dsAKH1; dsEGFP was used as a control (*n* = 5). (**B**) Movement trajectory after injection with dsAKH1. (**C**) Changes in motion distance. (**D**) Changes in moving time. (**E**) Changes in resting time. (**F**) Changes in active time. The data in (**A**,**D**,**E**) were analyzed using Student’s *t*-test. The data in (**C**,**F**) were analyzed using the Mann–Whitney U test.

**Figure 7:**
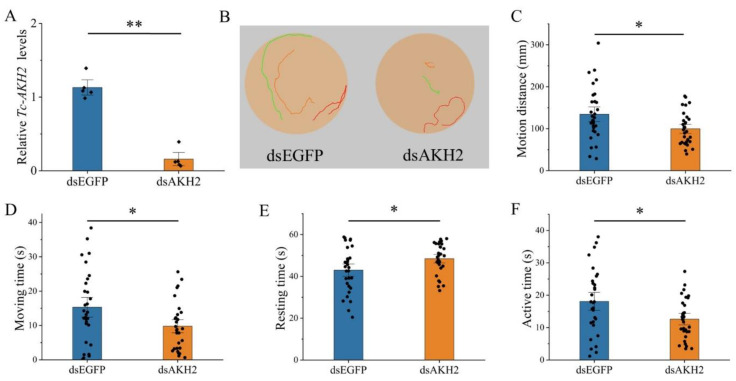
Effect of dsAKH2 injected into three-day-old adults on the gene transcript levels and motion situation of Tc-AKH2. Date are means ± SE. * *p* < 0.05, and ** *p* < 0.01. (**A**) Relative expression levels of Tc-AKH2 at 48 h after injection with dsAKH2; dsEGFP was used as a control (*n* = 5). The data were analyzed using the Mann–Whitney U test. (**B**) Movement trajectory after injection with dsAKH2. (**C**) Changes in motion distance. (**D**) Changes in moving time. (**E**) Changes in resting time. (**F**) Changes in active time. The data in (**C**–**F**) were analyzed using Student’s *t*-test.

To investigate the role of *Tc-AKHs* and *Tc-AKHR* in locomotor behavior, RNAi was employed to suppress their expression, followed by behavioral analysis in adult beetles. The knockdown efficiency was confirmed through qRT-PCR, revealing significant reductions in mRNA levels: *Tc-AKHR* by 81%, *Tc-AKH1* by 97%, and *Tc-AKH2* by 86% (Figure 5A, Figure 6A and Figure 7A). No cross-regulatory effects were observed between dsAKH1 and dsAKH2 (Figure S1).

The subsequent tracking of adult movement behavior demonstrated that the locomotor trajectories of RNAi-treated groups (dsAKHR and dsAKHs) were significantly shorter than those of the control group (dsEGFP) (Figure 5B, Figure 6B and Figure 7B). Further quantitative analysis indicated a marked change in key locomotor parameters, including total motion distance, movement duration, resting time, and active time, across all RNAi groups compared to controls (*p* < 0.05). Specifically, motion distance was significantly reduced with dsAKHR (91.73 mm, *p* < 0.01), dsAKH1 (153.28 mm, *p* < 0.01), and dsAKH2 (99.73 mm, *p* < 0.05) relative to dsEGFP controls (140.62 mm, 244.86 mm, and 134.57 mm, respectively) (Figure 5C, Figure 6C and Figure 7C). Similarly, movement duration was significantly decreased with dsAKHR (8.17 s, *p* < 0.05), dsAKH1 (18.53 s, *p* < 0.05), and dsAKH2 (9.80 s, *p* < 0.05) compared to the control groups (14.33 s, 29.17 s, and 15.30 s, respectively) (Figure 5D, Figure 6D and Figure 7D).

Furthermore, RNAi treatment significantly altered resting and active times. Resting times with dsAKHR (50.66 s, *p* < 0.05), dsAKH1 (39.99 s, *p* < 0.05), and dsAKH2 (48.45 s, *p* < 0.05) were significantly longer than those in controls (44.17 s, 30.64 s, and 42.98 s, respectively) (Figure 5E, Figure 6E and Figure 7E). Active time also exhibited significant reductions in dsAKHR (9.82 s, *p* < 0.01), dsAKH1 (10.16 s, *p* < 0.05), and dsAKH2 (12.60 s, *p* < 0.05) relative to control values (16.43 s, 17.62 s, and 18.10 s, respectively) (Figure 5F, Figure 6F and Figure 7F).

## 4. Discussion

In this study, two AKH genes and a single *AKHR* gene were identified in *T. castaneum*. In most insect species, one or two genes that encode AKH precursor genes have been identified, whereas, for instance, in *L. migratoria*, four AKH-encoding genes have been identified. So far, the evolutionary dynamics and functional divergence of AKH genes in regulating species-specific behavioral adaptations remain unknown. Also, it is unclear whether gene duplication leads to functional differentiation in *T. castaneum*. *Tc-AKHR* shares high similarity with AKHRs from other insect orders, including Lepidoptera and Diptera. Phylogenetic analysis confirmed that *T. castaneum AKHR* is orthologous to AKHRs in other insects. Functional calcium reporter assays showed that *Tc-AKHR*-transfected CHO cells responded to two AKH peptides in a concentration-dependent manner, consistent with previous findings in *D. melanogaster*, which confirmed the specificity of receptors for their corresponding ligands [22]. However, the use of CHO cells as a heterologous expression system raises inherent questions regarding the reconstitution of the endogenous cellular milieu of *T. castaneum*, which may bias receptor signal kinetics. Further research could use compatible *T. castaneum* cells. The *Tc-AKH1*, *Tc-AKH2*, and *Tc-AKHR* expressions were assessed in different tissues of adults according to data from the Beetle Atlas database. We found very high levels of *Tc-AKHs* only in the brain, which is not strange for a neuropeptide gene. For *Tc-AKHR*, high levels of expression were found in the fat body, which is the principal insect organ that stores both fat and sugar reserves, consistent with findings in other species [5,23,24,25]. The fat body is a key organ for lipid storage and energy regulation, and AKHR is known to mobilize lipids from this organ [26]. *Tc-AKHR* was also highly expressed in the carcass, suggesting its involvement in other physiological functions, such as reproduction, muscle tonus, and heart rate [27,28,29].

RNAi techniques were applied to suppress the expression of *Tc-AKH1*, *Tc-AKH2*, and *Tc-AKHR* in *T. castaneum*, resulting in decreases in locomotor activity. The silencing of *Tc-AKH1* or *Tc-AKH2* produced the same results in locomotion; therefore, we did not find evidence to support the notion that the gene duplication of AKH led to functional differentiation. It is hypothesized that the duplication of the AKH gene resulted in a compensatory effect on locomotion, potentially enhancing its adaptive significance. The influence of AKH on *T. castaneum*’s locomotion is likely attributed to its role in energy regulation. AKH specifically binds to its receptor, triggering enzymatic activation, including glycogen phosphorylase and triglyceride lipases, which facilitate energy mobilization [30,31]. The primary function of AKH is to induce lipid and carbohydrate release from the fat body. The lipid mobilization mechanism is mediated by AKH through triglyceride lipase [32,33]. In parallel, an alternative lipolytic pathway involves Brummer lipase, an enzyme homologous to mammalian adipose triglyceride lipase. *Drosophila* flies deficient in Brummer exhibit impaired lipid mobilization and excessive fat accumulation, while AKH responds to acute lipid demands. Brummer maintains basal lipid homeostasis and contributes to energy supply during metabolic exigencies [34,35]. AKH-mediated lipid mobilization is facilitated by intracellular signaling cascades [36]. One pathway involves calcium release from the endoplasmic reticulum, which acts as a crucial lipid mobilization signal. Another pathway entails AKH binding to its receptor on fat body cells, activating adenylate cyclase and increasing cyclic adenosine monophosphate (cAMP) levels. This cascade activates cAMP-dependent protein kinase A, which subsequently phosphorylates lipid droplets, triglyceride lipase, and perilipin 1/lipid storage droplet-1 protein [37]. Collectively, these findings indicate that AKH regulates peripheral energy reserves and locomotor activity via an intricate signaling network. Further research is necessary to unravel the molecular mechanisms behind this network.

In conclusion, the molecular characteristics of AKHs and AKHR in *T. castaneum,* along with their pharmacological properties, have been described. Through RNAi-mediated suppression of *AKHs* or *AKHR,* the pivotal role of AKH/AKHR signaling in locomotor activity has been confirmed. These findings not only strengthen the established function of AKH in locomotor control but also offer mechanistic insights into the neuromodulatory regulation of insect movement. This contributes to a more comprehensive understanding of neurohormonal pathways related to locomotion.

## Data Availability

The original contributions presented in this study are included in the article and supplementary material. Further inquiries can be directed to the corresponding author.

## Supplementary Materials

The following supporting information can be downloaded at: https://www.mdpi.com/article/10.3390/insects16040407/s1. Figure S1. (A) Effect of dsAKH1 injected into three-day-old adults on the gene transcript levels of Tc-AKH2. (B) Effect of dsAKH2 injected into three-day-old adults on the gene transcript levels of Tc-AKH1; Video S1. Motion video after injection with dsAKHR; Video S2. Motion video after injection with dsAKH1; Video S3. Motion video after injection with dsAKH2. Table S1. Primers of this study.
