# Supplementary material for: Adipokinetic Hormones and Their Receptor Regulate the Locomotor Behavior in Tribolium castaneum
Source: Insects. 2025 Apr 12;16(4):407. doi: 10.3390/insects16040407 (PMC12028090; doi:10.3390/insects16040407)
Supplement: Supplementary file 1 [file insects-16-00407-s001.zip › Figure S1.pdf]

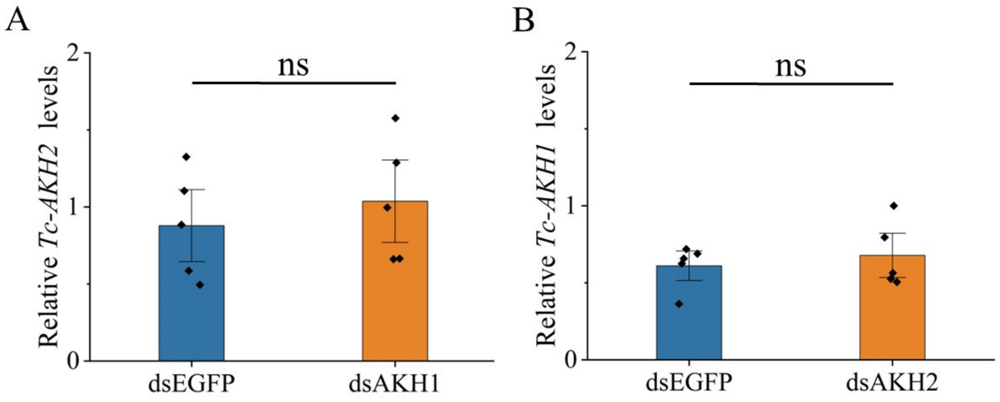


Figure S1. (A) Effect of dsAKH1 injected into three-day-old adults on the gene transcript levels of Tc-AKH2. (B) Effect of dsAKH2 injected into three-day-old adults on the gene transcript levels of Tc-AKH1.
