# Supplementary material for: Adipokinetic Hormones and Their Receptor Regulate the Locomotor Behavior in Tribolium castaneum
Source: Insects. 2025 Apr 12;16(4):407. doi: 10.3390/insects16040407 (PMC12028090; doi:10.3390/insects16040407)
Supplement: Supplementary file 1 [file insects-16-00407-s001.zip › Table S1.pdf]

Table S1. Primers of this study.

Primer Name	F-Primer Sequence (5′–3′)	R-Primer Sequence (5′–3′)	
dsAKHR	CCACCTACGCACACGAGTTGAC	CACCTTCCGTCCAGTCCGTCTA	dsRNA synthesis
dsAKH1	AGCTTTCGAGGATAAGGTGTACT	CGTTTCCACTAGGCGTAT	
dsAKH2	CCAACAGATGCAAAGAGTCC	CATGGGCATAAAATAAACGG	
dsEGFP	TAATACGACTCACTATAGGGTGG GCACAAATTTTCTGTC	TAATACGACTCACTATAGGGAAG GGTATCACCTTCAAAC	
AKHR	GGACCCAATGAGTGCTAG	CTTCGTTAAACCGCATACT	RT-qPCR
AKH1	CCGGTTCCGACGCAAATAACTG	AGGGCACACGAGTGATGAAACA	
AKH2	GTTTGTGTGCTGCCCAGTT	ATCTGTTGGATTCGCCTTCTG	
Rps3	TCAAATTGATCGGAGGTTTG	GTCCCACGGCAACATAATCT	
